# The Effect of the Conformation Process on the Physicochemical Properties of Carboxymethylcellulose–Starch Hydrogels

**DOI:** 10.3390/gels11030183

**Published:** 2025-03-06

**Authors:** Priscila Vedovello, Robert Silva Paiva, Ricardo Bortoletto-Santos, Caue Ribeiro, Fernando Ferrari Putti

**Affiliations:** 1 Embrapa Instrumentation, Rua XV de Novembro 1452, São Carlos 13560–970, SP, Brazil; pri.vedovello@gmail.com (P.V.); caue.ribeiro@embrapa.br (C.R.); 2 School of Agricultivation, São Paulo State University (UNESP), Rua José Barbosa de Barros 1870, Botucatu 18610-307, SP, Brazil; 3 UFSCar, Rodovia Washington Luiz, km 235, São Carlos 13565-905, SP, Brazil; robert21sp@gmail.com; 4 Postgraduate Program in Environmental Technology, University of Ribeirão Preto (UNAERP), Avenida Costábile Romano, 2201, Ribeirão Preto 14096-900, SP, Brazil; 5 School of Sciences and Engineering, São Paulo State University (UNESP), Rua Domingos da Costa Lopes 780, Tupã 17602-496, SP, Brazil

**Keywords:** biomaterial, polymer, solvent casting, extrusion, morphology, porosity

## Abstract

This study discusses the preparation of biopolymeric hydrogels (a biomaterial) via different techniques, such as casting and extrusion, to compare the effects of the process and the use of citric acid as a crosslinker on the morphology, physicochemical properties, and degree of swelling of the hydrogel. Casting is widely used for its low cost and space-saving nature, but upscaling is problematic. Extrusion offers a way to produce materials in large quantities; these materials can undergo mechanical and thermal energy, which can significantly alter their properties. The samples obtained by extrusion had porous surfaces, which are critical for the water penetration and swelling of superabsorbent hydrogels. In contrast, the hydrogels produced by casting did not form pores, resulting in a lower degree of swelling. Extrusion increased the degree of swelling threefold due to the formation of pores, influencing water absorption and diffusion dynamics, especially in samples with higher starch content, where crosslinking occurred more effectively.

## 1. Introduction

Agricultural development is driven by the need to increase crop productivity due to population growth and the rising demand for food. Historically, these challenges have been mitigated by the intensive use of pesticides and fertilizers, as well as the overexploitation of natural resources, such as soil and water [1,2,3,4]. However, the issues of soil salinization and desertification, exacerbated by droughts and water scarcity, pose significant threats to sustainable agriculture. Therefore, developing innovative and sustainable irrigation techniques has become essential for ensuring long-term agricultural viability [5].

Hydrophilic substances, commonly called hydrogels, are promising for agricultural applications due to their unique water retention properties [6]. Research indicates that the application of hydrogels improves soil parameters, such as hydraulic conductivity [7], field capacity [8], and water retention [9], while also promoting improved crop development [10,11,12].

Hydrogels based on biopolymers such as starch (S), carboxymethylcellulose (CMC), and gelatin, among others, have been obtained through different processes, such as casting, extrusion, injection, or thermo-pressing [13,14,15,16]. According to Madhumitha, G. et al. (2018) [17], most studies use the casting method on a laboratory scale to create materials of this type due to the cost benefits of the technique and limited space. Nevertheless, a significant issue with the casting approach is the high processing cost associated with the low production volume and the challenge of upscaling it to an industrial level [18,19].

Studies describing the utilization of procedures such as extrusion are scarce despite the literature having a large amount of research on hydrogel manufacturing utilizing the casting approach [19]. Extrusion exposes materials to mechanical and thermal energy, which causes chemical and physical reactions that significantly affect the material’s final properties [20]. This technique offers a highly effective solution for meeting high demands at a minimal cost. Extrusion stands out as the predominant method for producing industrial plastics on a large scale, owing to its ability to rapidly reach high temperatures and its straightforward operation [19].

The objective of the present study was to evaluate the effect of the processing technique (casting and extrusion) on the morphology, physicochemical properties, and degree of swelling of CMC hydrogels crosslinked with citric acid.

## 2. Results and Discussion

### 2.1. Characterization

**Figure 1:**
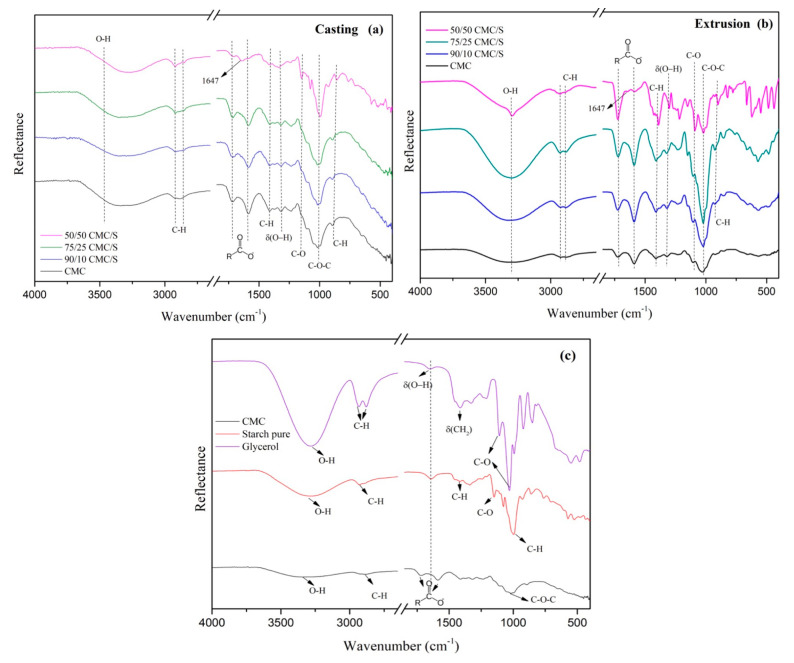
FTIR spectra of samples: (**a**) casting, (**b**) extrusion, and (**c**) pure components.

The Fourier transform infrared (FTIR) spectra provide details about how the functional groups of the various components interact. Figure 1 shows the FTIR spectra of CMC, starch, glycerol, and CMC/starch/glycerol.

The vibrational infrared absorption spectrum of glycerol P.A. presents a broad absorption band at 3281 cm^−1^, characteristic of the n(O–H) stretching originating from alcohol groups. The 2934 and 2879 cm^−1^ bands can be attributed to the n(C–H) bond stretching mode. The absorption at 1654 cm^−1^ corresponds to the angular deformation of the δ(O–H) group. The absorption band at 1407 cm^−1^ may be related to the angular deformation δ(–CH_2_), and absorptions at 1104 and 1029 cm^−1^ correspond to the stretching n(C–O) of secondary and primary alcohol, respectively [21].

The pure CMC spectrum presents a band at 2917 cm^−1^ referring to asymmetric n(C–H) stretching, and a broad band located around 3590–3039 cm^−1^ is attributed to n(O–H) stretching [22]. The peaks at 1588 and 1715 cm^−1^ are associated with the vibrational stretching movements, both symmetric and asymmetric, of the carboxylate groups, respectively [22]. n(C–O–C) vibration stretching, referring to the polysaccharide skeleton, can be observed at 1041 cm^−1^ [23], and the δ(O–H) angular deformation is found at about 1316 cm^−1^ [24].

In the spectrum of pure starch, a representative peak is found at 1644 cm^−1^ due to the presence of absorbed water. The peak at 1160 cm^−1^ arises from the n(C–O) stretch, and the n(C–H) stretch is found at 2929 cm^−1^ [24]. Due to intermolecular and intramolecular hydrogen bonds and free -OH groups, a broad band is found at 3310 cm^−1^ related to n(O–H) stretching vibrations. The peak at 1453 cm^−1^ is attributed to the in-plane C–H bending of the CH_2_ groups, and the one at 994 cm^−1^ is attributed to the out-of-plane band [25].

**Figure 2:**
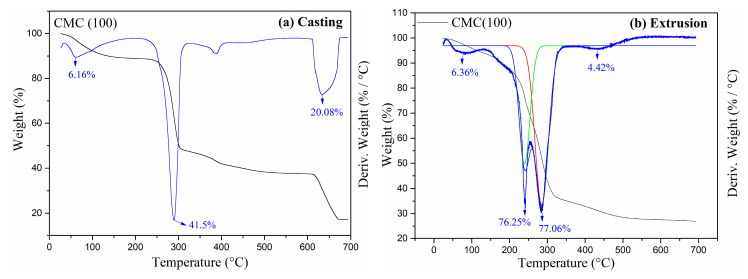
TGA (black color) and derived thermogravimetric analysis (DTG) (blue color) of CMC: (**a**) casting and (**b**) extrusion.

The intense band found between 1064 and 955 cm^−1^ probably resulted from overlapping three-band characteristics of starch and cellulose [24]. Around 955 cm^−1^, it is attributed to the n(C–O) of the C–O–H group. The characteristic range of angular deformation of δ(C–H) is observed in the region of 880 cm^−1^. In the spectrum of the CMC–starch mixture, this band is shifted to 859 cm^−1^, and the band related to the carboxylate group in CMC is shifted to 1644 cm^−1^ (casting) and 1647 cm^−1^ (extrusion), indicative of ester bond formation between the hydroxyl groups in the amylopectin branches of the starch and the carboxylic acid groups of the CMC, forming a stable crosslinked structure between the starch and the CMC, in which it is possible to observe a band close to 1719 cm^−1^, related to the n(C=O) stretching of the carbonyl ester [24,26]. The n(C–O–C) stretching vibration of the polysaccharide skeleton is also changed to 1000 cm^−1^ [27]. The absence of differences observed in the FTIR spectra between the casting and extrusion procedures was likely due to the starch molecules’ non-drastic alterations, which were too subtle for this approach to detect. Figure 2 presents the findings of thermogravimetric analysis (TGA) of the samples’ thermal stability.

The formation of CMC by casting and extrusion was analyzed using thermal gravity analysis (TGA), as shown in Figure 2. Three different weight decreases are seen. The first weight loss occurs at 60 °C for both processes, or approximately 6.16% and 6.36%. The sample’s first weight loss can be attributed to moisture. Rani et al. [27] reported a similar observation. This is a result of biopolymers’ propensity to take in moisture from their immediate environment [27].

There is a second weight maximum degradation with a weight loss of 41.5% at 289.74 °C, which corresponds to the loss of COO- from the polysaccharide; in this temperature range, it undergoes decarboxylation [28]. In the extruded sample, this mass loss occurs in two stages (according to deconvolution), with losses of 76.25% and 77.06%, at 242.46 °C and 284.07 °C. The third mass loss for the sample obtained by casting started around 608.55–675.76 °C, with a maximum of 633.36 °C, resulting in a weight loss of 20.08%. However, for the extruded sample, there was a low mass loss, 4.42% at 433.5 °C [29]. At 700 °C, CMC presented residual weights of 17.26% and 27.09%, respectively, for the casting and extrusion samples, indicating the presence of a non-volatile component fraction [30].

**Figure 3:**
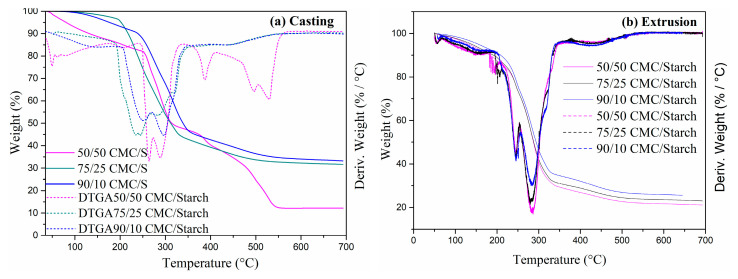
TGA and DTG analysis of CMC/S: (**a**) casting and (**b**) extrusion.

Thermogravimetry was employed to assess the thermal stability of blends of starch and CMC, the result of which is shown in Figure 3. Furthermore, the material’s thermal decomposition temperature was determined in four main steps using the derivative of the TGA curves. About 89 °C was the first degradation temperature of the 50/50 CMC/S-containing material, which corresponds to water loss; 207 °C is the second stage of the thermal degradation of the samples, which is related to the volatilization of glycerol; at 262, 288, and 386 °C is the third stage; and the fourth stage at 527 °C corresponds to the degradation of starch and CMC constituents [31]. For the 50/50 CMC/S sample, the CMC presented residual weights of 12.27% at 700 °C [30].

The degradation zones overlapped in the 75/25 and 90/10 CMC/S thermograms. The initial degradation peak, corresponding to the glycerol volatilization, occurred at 250 °C and 237 °C for 90/10 and 75/25 CMC/S, respectively. At 319 °C and 285 °C, the starch and CMC components were degraded to 75/25 and 298 °C to 90/10 CMC/S [31]. However, for the samples 75/25 and 90/10 CMC/S, the CMC presented residual weights of 31.7% and 33.30%, respectively, at 700 °C, also suggesting greater stability, obtaining a greater fraction of the non-volatile component [30].

The extruded samples maintained the same degradation profile. Thus, there was also an overlap in the degradation zones in the thermograms. The initial degradation peak occurred at around 245 °C. The second degradation peak occurred around 283 °C, corresponding to the starch and CMC components [31]. The residual mass of these samples was around 21.6%, 23.10%, and 26.21% for 50/50, 75/25, and 90/10 CMC/S, respectively, due to the non-volatile fraction of CMC [30].

**Figure 4:**
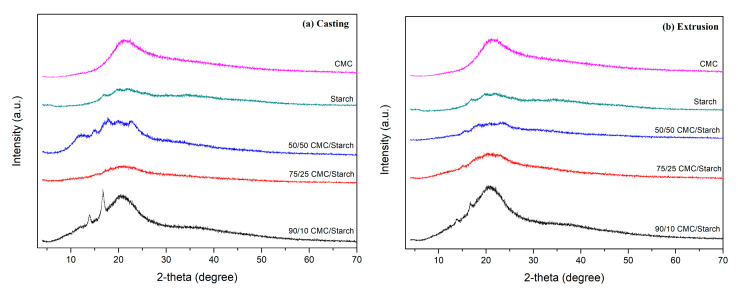
X-ray diffraction (XRD) patterns of CMC, starch, and their respective formulations: (**a**) casting and (**b**) extrusion.

Figure 4 presents the X-ray diffraction patterns of CMC, starch, and their respective formulations. The starch diffractogram shows an amorphous halo superimposed on intense diffraction peaks, indicating the polymer’s semicrystalline state. Moreover, the starch diffractogram presented peaks at 17.3, 18.2, and 23.1°, characteristic of the type A polymorphic structure of amylose and amylopectin. In the same context, the CMC shows a broad peak at about 2θ = 22.4°, indicating that its crystal structure was amorphous. The peaks for the different formulations were similar, and the main peaks were observed between 15 and 25°. It is possible to verify that the characteristic peaks for the formulations (casting and extrusion) coincide with the diffraction signals for the precursor reagents, such as starch and CMC. These observations suggest that the diffractograms are a superposition of pure starch and CMC profiles. The characteristics of the peaks also show no crystalline transformations of the structure based on the treatments.

### 2.2. Mechanical Properties

**Figure 5:**
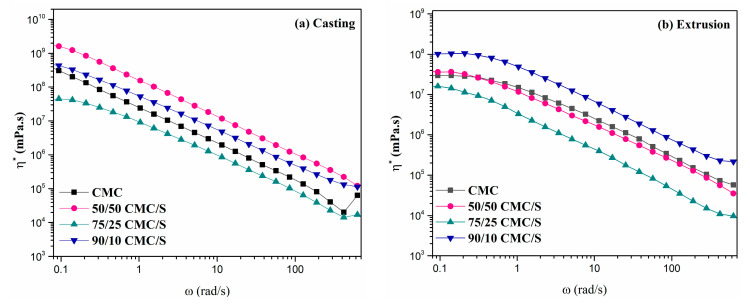
Complex viscosity versus angular frequency for samples with varying CMC/S ratios was prepared using (**a**) casting and (**b**) mechanical extrusion.

Figure 5 presents the viscosity versus angular frequency results for samples with varying CMC/S ratios, prepared using two different methods: (a) casting and (b) mechanical extrusion. As observed in both the casting and extrusion samples, viscosity decreases with increasing shear rate, confirming the pseudoplastic fluid behavior described in the literature [19,32]. However, notable differences were observed, including the profile of the viscosity decrease curve during the frequency sweep, the viscosity values, and the influence of mixture proportions depending on the methodology used to produce the samples.

A significant decrease in viscosity is observed for the CMC/S mixtures produced by casting as the shear rate increases, with a slight Newtonian plateau at low frequencies for the 75/25 ratio sample. This suggests that this production method results in structures with a low degree of organization. In contrast, samples produced by extrusion exhibit a Newtonian plateau across all formulations, followed by a gradual decrease in pseudoplastic behavior as the shear rate increases. This indicates that the material produced through extrusion has a higher degree of chain organization [32].

The 50/50 and 90/10 CMC/S ratio samples showed increased viscosity for casting and extrusion samples. In the casting method, the highest viscosity increase occurred in the 50/50 sample, while in the extrusion method, the greatest increase was seen in the 90/10 sample compared to pure CMC obtained from both methods. Additionally, the 75/25 ratio sample showed a decrease in viscosity for both production methods, with a result lower than that of the pure CMC sample.

In general, the methods used to obtain the CMC/S mixtures showed the same degree of increase in viscosity for the 50/50 and 90/10 samples and the same tendency to decrease for the 75/25 sample compared to the control sample, CMC. However, the methods differed regarding the chains’ organizational structure, as shown by the Newtonian plateau, which was more pronounced for the samples produced by the extrusion process, as described above. This corroborates the degree of swelling since the higher the viscosity degree, the greater the diffusivity of water molecules through the polymer chains.

### 2.3. Morphology

**Figure 6:**
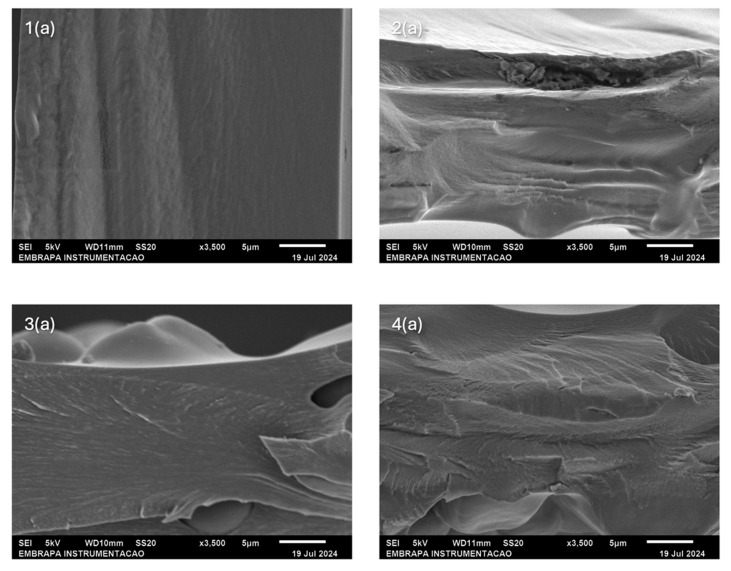

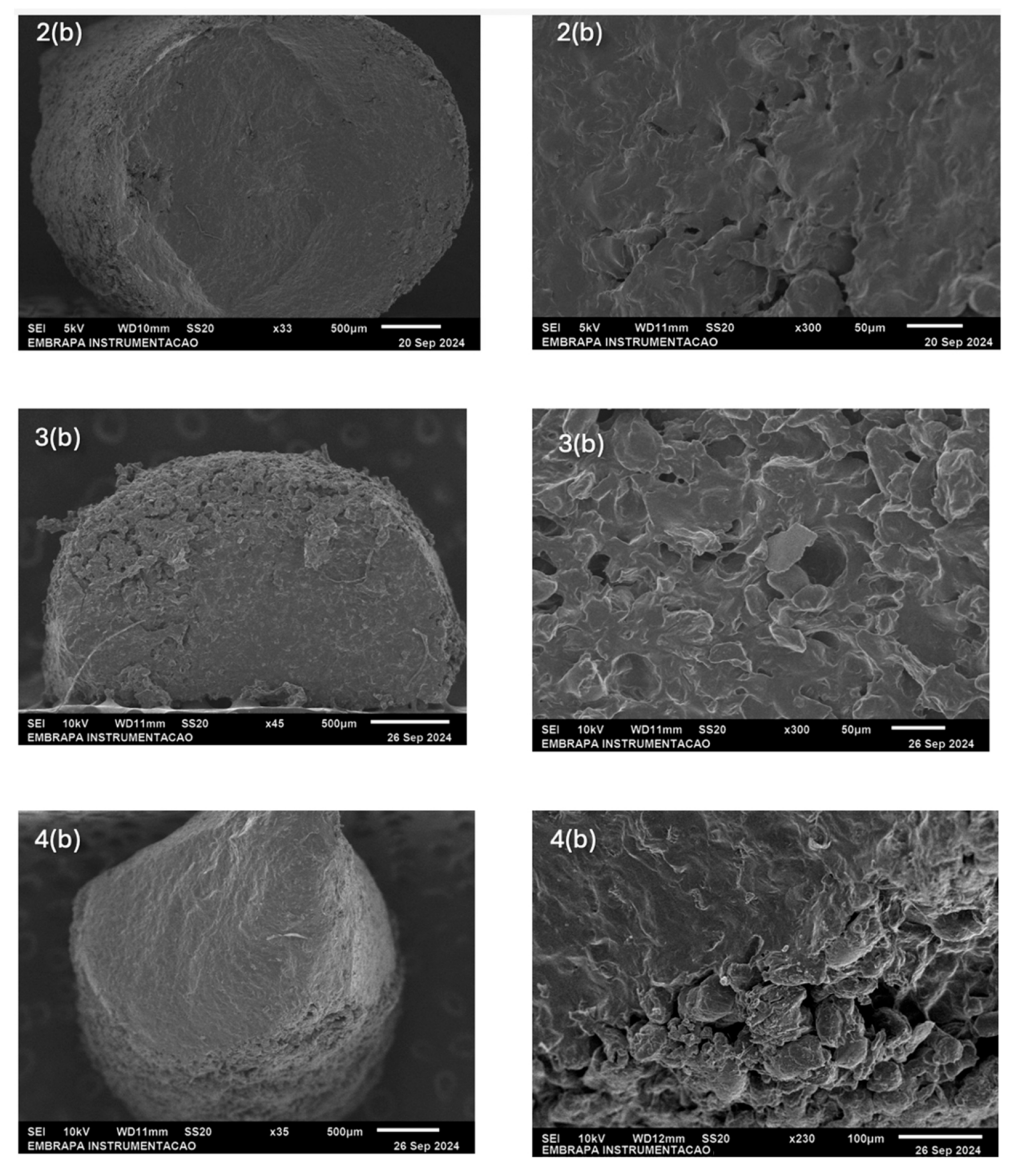
The SEMs of the cryogenic fracture surface of the composites obtained by (**a**) casting and (**b**) extrusion: 1. CMC pure; 2. 90/10 wt. CMC/S; 3. 75/25 wt. CMC/S, and 4. 50/50 wt. CMC/S.

Figure 6 shows the scanning electron micrographs (SEMs) of the cryogenic fracture surface micrographs of the composites obtained by casting (a) and extrusion (b). The films obtained by starch casting and CMC exhibited homogeneous and compact structures without pores.

Under magnification, the samples obtained by extrusion (Figure 6 (2b–4b)) show many pores distributed heterogeneously along their surface, most of which are interconnected. The pores allow water to penetrate the structure of the superabsorbent hydrogels used in agriculture, and their presence is essential to favor swelling. Duquette et al. [33] report that the surfaces of hydrogels made of starch and citric acid created in an aqueous medium are highly porous and irregular. These pores allow the diffusion of solvents and ions within the hydrogel matrix and facilitate particle trapping.

In contrast to samples produced by extrusion, the casting process (Figure 6 (1a–4a)) produces “dense hydrogels”. Although the literature uses dense hydrogel terminology, these materials allow for the easy diffusion of water molecules with dimensions of 0.265 nm. In non-porous hydrogels, swelling is controlled by the diffusion of water within the polymer network. Meanwhile, open-pore hydrogels can induce capillary action driven by surface tension [34]. Therefore, porous hydrogels tend to permeate more water molecules. This causes the degree of swelling of a non-porous hydrogel to be lower, as demonstrated by the swelling tests, which proved that samples obtained by casting presented a lower degree of swelling.

Extrusion is more advantageous in industrial processes that require high productivity and low operating costs due to several factors that optimize efficiency and reduce costs. Unlike casting, which occurs in batches, extrusion is a continuous process, allowing uninterrupted production. This reduces waiting times and increases production volume in less time. The energy consumption per part produced is lower since the continuous process avoids repetitive heating and cooling cycles. Extrusion ensures uniformity in the final product, which is essential for industrial applications that require dimensional accuracy and structural consistency.

### 2.4. Degree of Swelling

**Figure 7:**
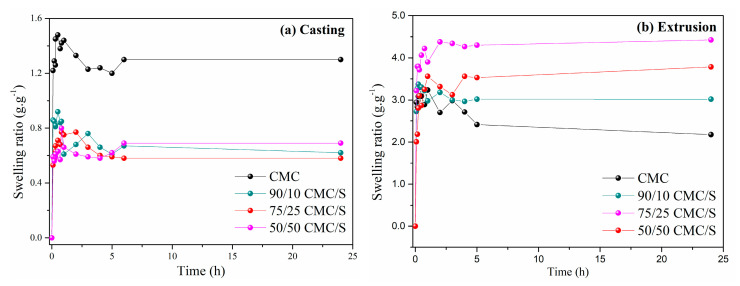
The swelling ratio of CMC/S: (**a**) casting and (**b**) extrusion.

The degree of swelling of the 100% CMC for the sample processed by casting showed a rapid increase up to 30 min of analysis, then a small decrease until equilibrium, around 180 min, after immersing the material in distilled water. Samples containing CMC/S showed a lower degree of swelling throughout the process when compared to pure CMC (Figure 7a). However, the casting method used to process the hydrogels showed a low swelling rate.

Extrusion processing achieved three times greater degrees of swelling than casting processing, particularly for samples with higher starch concentrations (Figure 7b). This implies that crosslinking occurred more effectively during processing. This increase in the swelling degree is due to the presence of pores in the extruded samples. The existence of pores in it can significantly alter a polymeric material’s characteristics [34].

A hydrogel swells due to a complex interaction between chemical and physical factors, allowing liquid to seep into the polymer matrix. Hydrophilic groups first absorb water in the hydrogel due to diffusion forces. Consequently, the selection and ratio of material crosslinking play a crucial role in dictating the dynamics of the diffusion process. In the early stages, water is rapidly absorbed owing to the substantial free space between the crosslinked chains. However, as the process continues, the rate of water sorption decreases because molecules encounter barriers at the crosslinking points, hindering further absorption and leading the material toward equilibrium [26,35].

Several factors, such as crosslinking density, chemical composition, and porosity, act to ensure that the hydrogel reaches equilibrium, all of which govern characteristics such as hydrophilicity, charge, and intermolecular interactions [26,36]. Images of the samples before and after swelling are contained in the supplementary mate-rial (Figure S1 and Figure S2).

### 2.5. Porosity Measurement

**Figure 8:**
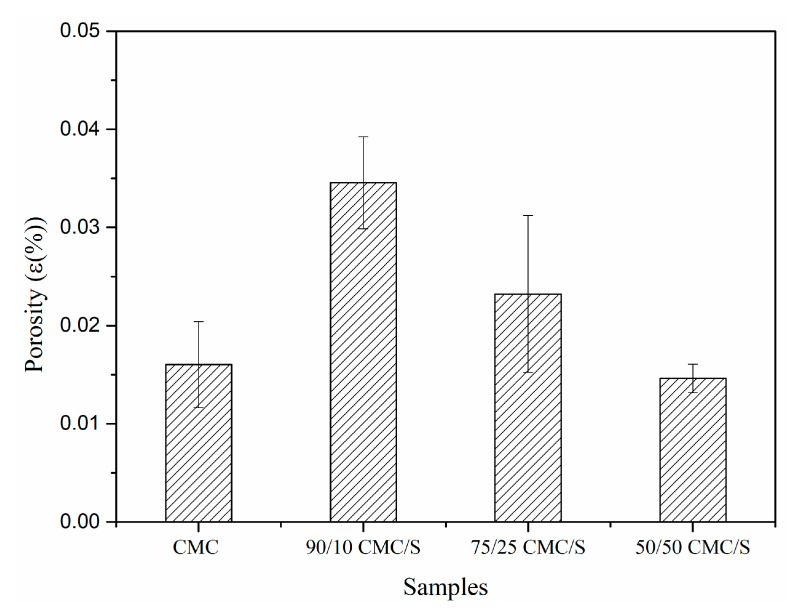
The percentage of the porosity of the samples obtained by extrusion.

Figure 8 contains the porosity values of the hydrogels obtained by extrusion. The porosity of these hydrogels varied from 0.16 to 0.34% because pore formation occurred mainly on the surface. When S/CMC is processed, these compounds combine inside the extruder, increasing viscosity through cross-linking. This affects the stages of air bubble nucleation and bubble growth, predominantly on the hydrogel’s surface, where pores can be seen forming, as seen in SEM images [26].

The viscoelastic matrix’s properties influence the expansion and porosity of the materials based on extruded starch. Both expansion and porosity depend on the capacity of materials to resist the pressure that water vapor exerts, which is the result of superheated water evaporating after the molten material exits the matrix [37]. Karathanos, V.T. and Saravacos, G.D. [38] suggest that extrusion processing affects the porosity of starch materials since they are susceptible to factors such as the cylinder temperature, screw speed, and moisture content of the feed material [38]. As can be seen, with the addition of starch, there is an increase in porosity in relation to pure CMC. However, there is a threshold for this concentration when compared to the samples containing the polymer blend. When increasing the starch concentration, there was a decrease in porosity, possibly due to interactions between the CMC and starch chains; both polymers have hydroxyl groups (-OH), which allows the formation of extensive hydrogen bond networks between CMC and starch, promoting the stability of the mixture and influencing viscosity and water retention.

## 3. Conclusions

This study emphasizes the notable distinctions between the characteristics of hydrogels made by casting and extrusion techniques, especially when crosslinking with citric acid. The extrusion procedure created highly porous materials that permitted enhanced water absorption and swelling, whereas the casting method produced denser hydrogels with lesser swelling capacities due to limited porosity. Processing methods enhance the hydrogels’ physical and chemical properties, and these features are critical for agricultural applications that aim to improve soil health and water retention. Further optimization of these hydrogels could provide viable solutions to address issues like soil degradation and water scarcity as the need for sustainable agricultural techniques rises.

## 4. Materials and Methods

### 4.1. Reagents

Carboxymethylcellulose (Dynamica^®^) CAS-9004-32-4Visc.1000/2000 CP, Commercial Starch—food grade (Amilogil^®^ 2100-Cargill^®^), Anhydrous Citric Acid PA (Êxodo científica^®^) CAS-77-92-9, Glycerol (Dynamica^®^) CAS-56-81-5.

### 4.2. Preparation of Hydrogels with Carboxymethylcellulose (CMC) and Starch (S) by Casting

The hydrogel synthesis was based on the work of De Albuquerque, G. et al. (2019) [39]. The films formed by the mixes were obtained using the solvent evaporation technique (casting), as indicated in Table 1. The polymers were dissolved in deionized water under mechanical stirring (1000 rpm) for two hours. Citric acid was added to the solution to promote crosslinking, and glycerol was used as a plasticizer (fixed to 5% in weight—wt%). The solutions were poured into Petri dishes for solvent evaporation at 100 °C to promote crosslinking (overnight).

### 4.3. Preparation of Hydrogels with Carboxymethylcellulose (CMC) and Starch (S) by Extrusion

The synthesis of hydrogels was based on the work of Cagnin, C. et al. (2021) [26]. The samples were produced by reactive extrusion, using citric acid as a crosslinker. The formulations are presented in Table 1.

**Figure 9:**
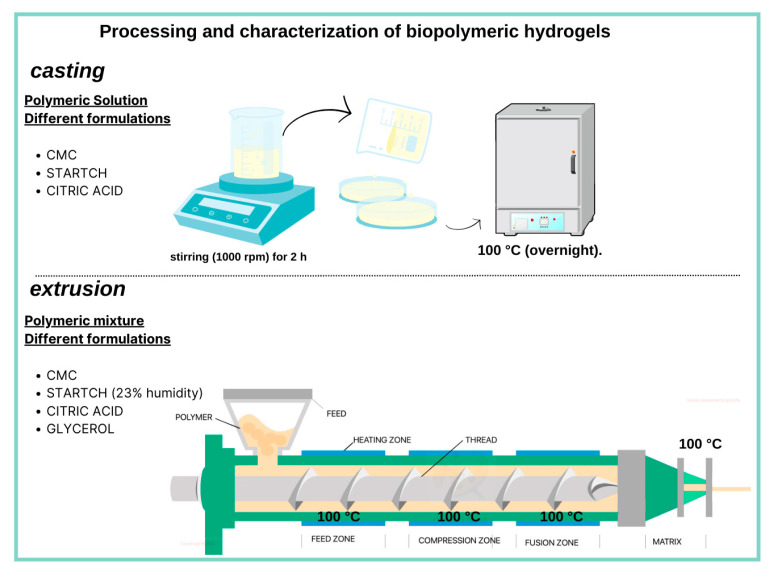
Schematic diagram of the different manufacturing processes of hydrogels. Created by Canvas^®^.

Starch-/CMC-containing hydrogels were prepared by mixing the components with enough water to condition the sample to 23% humidity and Glycerol (40% wt.) was used as a plasticizer. The samples were placed in airtight polyethylene bags for homogenization and given at least 12 h to equilibrate under refrigeration. The samples were allowed to reach 25 °C before processing. Extrusion was carried out in a Thermo Scientific Process parallel twin-screw extruder (Waltham, MA, USA) with cylinder length L = 40 D, and a standard configuration dies using 11 mm diameter pressure screws. The temperature profile, from the feed zone to the outlet zone, was 90, 100, 100, and 100 °C, and the screw rotation was 30 rpm. Figure 9 presents a schematic diagram of the different hydrogel manufacturing processes.

### 4.4. Fourier Transform Infrared Spectrometry (FTIR)

Fourier transform infrared (FTIR) analyses were performed using a Vertex 70 instrument (Bruker, Billerica, MA, USA) fitted with a diamond crystal in attenuated total reflectance (ATR) mode in the frequency range from 4000 to 400 cm^−1^, with 4 cm^−1^ resolution.

### 4.5. Thermal Properties

The thermogravimetric analysis was performed using a simultaneous thermogravimetric analyzer (TGA/DSC) (TA Instrument model SDT 650, New Castle, DE, USA), using ~10 mg of samples heated from 25 °C to 700 °C at a rate of 10 °C min^−1^ under nitrogen flow.

### 4.6. Reological Measurements

The rheological measurements were performed on an Anton Paar 302 (Graz, Austria) at 90 °C, using 25 mm diameter plates with a gap distance of 1 mm, under an inert nitrogen atmosphere. Complex viscosity (ƞ*) was measured via dynamic frequency sweep tests performed in the linear viscoelasticity regime at 0.1% strain amplitude within the angular frequency range 0.1–500 rad/s.

### 4.7. Scanning Electron Microscopy

All samples were frozen and fractured under liquid nitrogen to study their morphology. Scanning electron microscopy (SEM) was used, and the SEM analyses were carried out using the JEOL JSM 6510 equipment with an energy-dispersive X-ray analysis system. The samples were placed on a carbon tape fixed to a metal support (stub) surface and coated with gold in an ionization chamber (Baltec Med. 020). The SEM images were obtained with an accelerating voltage of 5 kV using a secondary electron detector of the JSM 6510 instrument (JEOL, Tokyo, Japan).

### 4.8. X-Ray Diffraction

The materials were characterized by X-ray diffraction (XRD) using a LabX XRD-6000 diffractometer (Shimadzu, Japan), operating at 30 mA and 30 kV. Each sample was placed in sample holders with a diameter and depth of 25 and 1 mm, respectively. Scans were performed in the 2θ = 5–70° range with Cu-Kα radiation of 1.5406 A° and a scanning rate 1.0° min^−1^.

### 4.9. Degree of Swelling

The swelling degree (SD) was determined as described by Cagnin, C. et al. (2021) with adaptations [26]. The samples were weighed to determine dry mass and then immersed in approximately 30 mL of water at 25 °C. At specific time intervals, the sample was removed, the excess solution was removed with absorbent paper for the casting samples, and the sample was weighed again.

For the extrusion samples, the swelling at different times was calculated following the protocol outlined by Yoshimura, T et al. (2006) and Marim, B.M. et al. (2022), using the tea bag method to measure the product’s water absorbency [40,41]. Within permeable nylon tea bags measuring 500 mm by 700 mm, dried pellets (0.5 g) were allowed to swell. The water was 25 °C, and the tea bag was immersed. After an hour of procedure, the tea bag was removed from the water, and any remaining water was drained for a minute. The swelling degree was calculated by Equation (1).

(1) $$SD=\frac{w_{S}-w_{i}}{w_{i}}$$

where *w_S_* is the mass of the wet sample, and *w_i_* is the initial dry mass.

The tea bag’s swollen weight (W_tb_) was also calculated (Equation (2)). The analyses were performed in triplicate.

(2) $$SD=\frac{w_{S}-{w_{tb}-w}_{i}}{w_{i}}$$

where W_s_ is the weight of the dry superabsorbent hydrogel sample, and W_tb_ is the weight of the tea bag following the water processing. The tea bag was immersed for two hours and lifted for one minute to test the absorbency again. The same method was used to assess the abstinence after 24 h.

### 4.10. Porosity Measurements

The porosity *ε* (%) of the samples was measured according to a gravimetric method, as in Equation (3) [42,43]:

(3) $$\varepsilon (\%)=\frac{W_{w}-W_{d}}{V\times \rho_{w}}\times 100$$

where (i) *W_w_* and *W_d_* are the masses of the samples: wet (immersed in hexane for 24 h) and dry (dried at 40 °C), respectively; (ii) *V* is the volume (cm^3^) of the samples; and (iii) *ρ_w_* is the density of hexane (0.659 g cm^−3^ at 20 °C). The volume of the samples can be measured from its dimensions, as it is cylindrical [43].

## Figures and Tables

**Table 1:** Sample formulation (g 100 g^−1^).

Sample	CMC	Starch	Citric Acid
CMC	100	0	20
CMC/S	90	10	20
CMC/S	75	25	20
CMC/S	50	50	20

## Data Availability

Data available on request.

## Supplementary Materials

The following supporting information can be downloaded at: https://www.mdpi.com/article/10.3390/gels11030183/s1, Figure S1: Swelling image of the samples by extrusion; Figure S2: Swelling image of the samples by casting.
