# Supplementary material for: The Effect of the Conformation Process on the Physicochemical Properties of Carboxymethylcellulose–Starch Hydrogels
Source: Gels. 2025 Mar 6;11(3):183. doi: 10.3390/gels11030183 (PMC11942467; doi:10.3390/gels11030183)
Supplement: Supplementary file 1 [file gels-11-00183-s001.zip › gels-3488331-supplementary.pdf]

Supplementary material

Annex 1 – Swelling image of the samples.

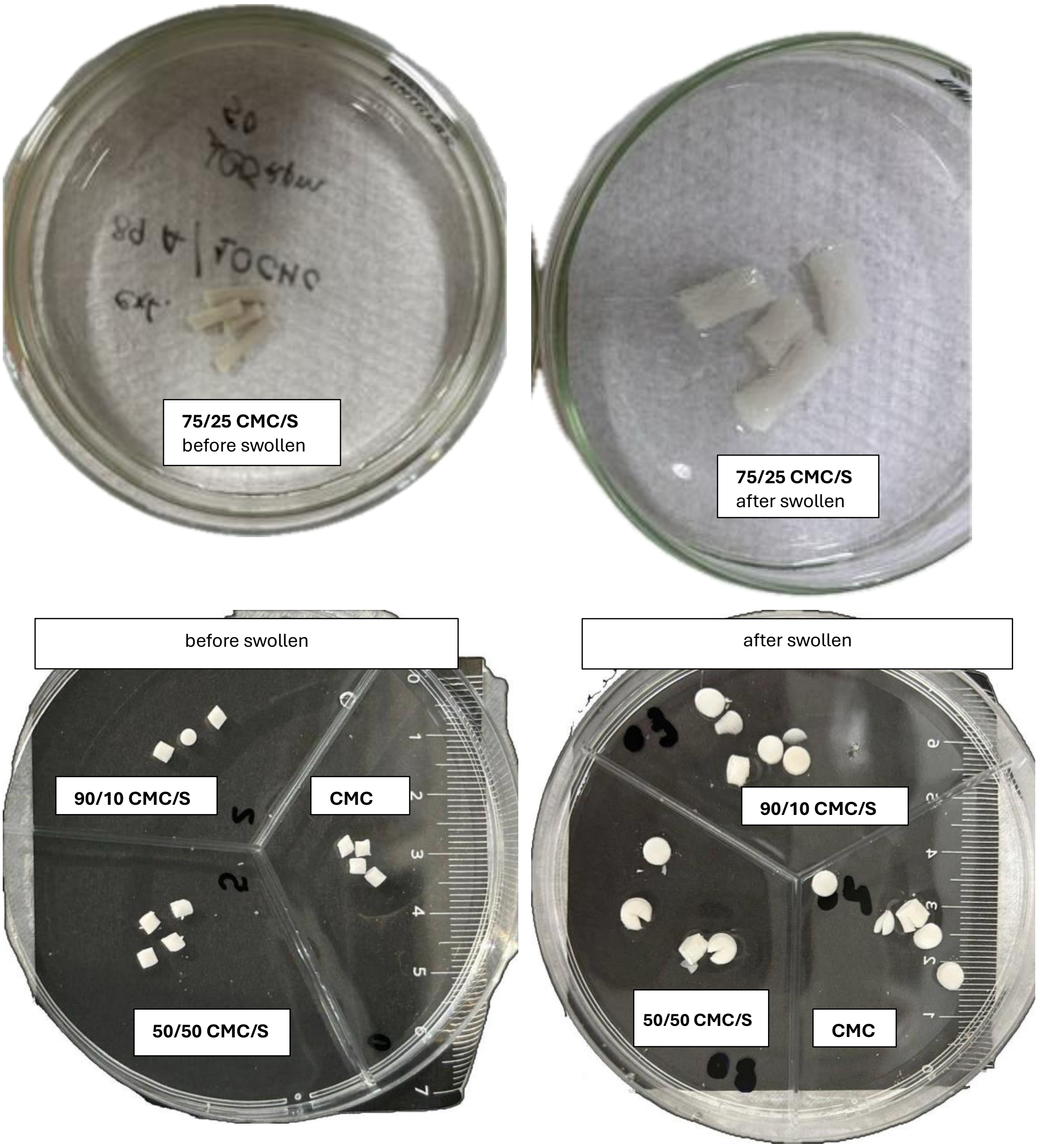

Figure S1 - Swelling image of the samples by extrusion.

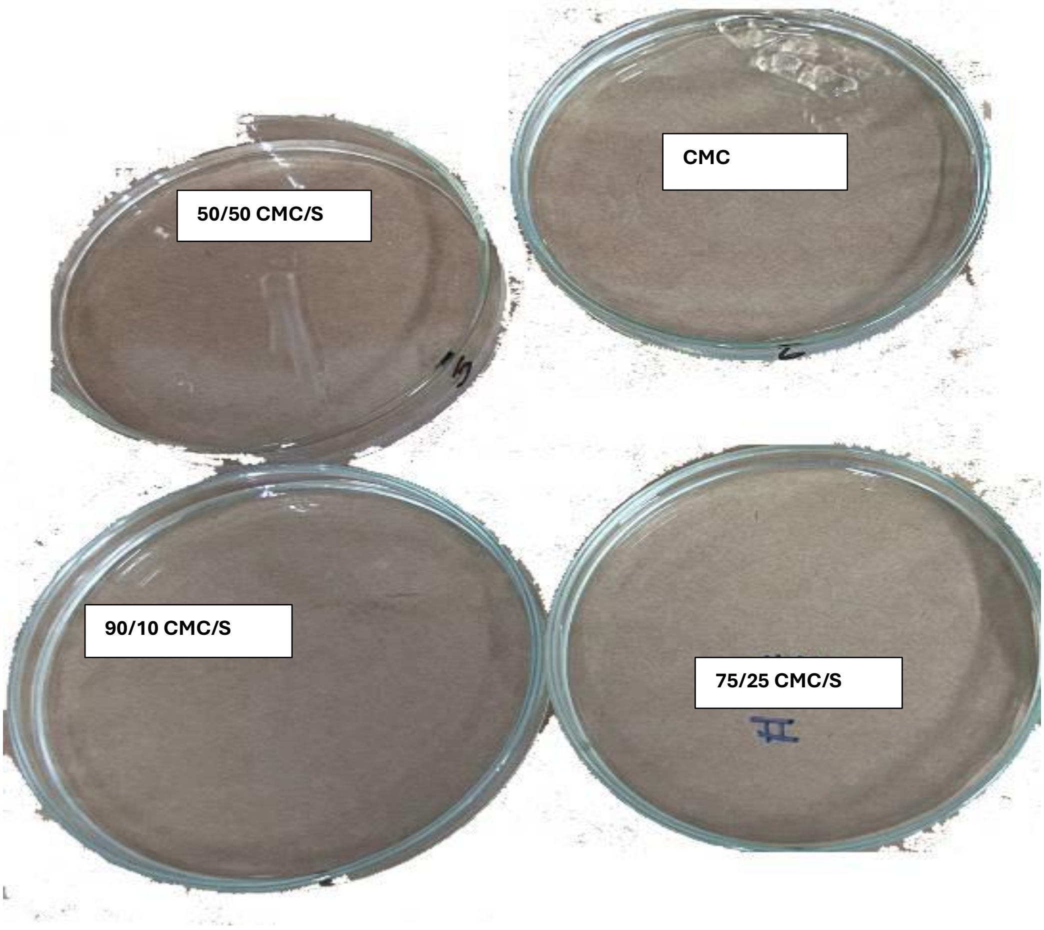

Figure S2 - Swelling image of the samples by casting.
